# Supplementary material for: Effect of Fermentation Humidity on Quality of Congou Black Tea
Source: Foods. 2023 Apr 20;12(8):1726. doi: 10.3390/foods12081726 (PMC10138149; doi:10.3390/foods12081726)
Supplement: Supplementary file 1 [file foods-12-01726-s001.zip › foods-2317632-supplementary.pdf]

Effect of Fermentation Humidity on Quality of Congou Black Tea

Supplementary

Table S1. Effect of fermentation humidity on the chromatic aberration of congou black tea's infusion.

Samples	L*	a*	b*	b*/a*
RH55	84.18±0.44a	6.16±0.53a	68.4±1.3a	11.15±0.78a
RH65	84.12±0.35a	6.16±0.47a	68.24±1.28a	11.11±0.63a
RH75	84.29±0.55a	5.8±0.7a	67.13±1.57a	11.68±1.19a
RH85	84.19±0.62a	5.8±0.69a	66.71±2.85a	11.65±1.83a
RH95	84.1±0.64a	5.94±0.69a	67.26±1.79a	11.4±1.04a

Note: L*, the brightness of the infusion; a*, red-green tones, with positive values indicating the degree of redness; b*, yellow-blue tones, with positive values indicating the degree of yellow; b*/a*, the hue. Different lowercase letters in the same column indicate a significant difference at $p < 0.05$.

Table S2. Content of volatile compounds under different fermentation humidity conditions. (μg/g)

RT	RI	Compound Name	RH55	RH65	RH75	RH85	RH95
		**Alcohols**					
4.30	863	3-Hexen-1-ol	6.07±0.23	6.05±0.25	6.56±0.64	7.67±1.91	7.74±0.81
8.09	1034	Benzyl alcohol	20.87±1.49	21.30±1.93	21.73±2.16	22.15±2.29	22.14±0.72
9.35	1073	cis-Furan linalool oxide	20.20±1.98	20.25±0.91	21.50±0.57	21.52±1.57	21.55±0.93
9.93	1089	trans-Furan linalool oxide	31.52±1.48	31.59±1.64	31.62±1.25	31.86±1.54	32.21±1.17
10.43	1102	Linalool	31.63±0.62b	31.87±1.59ab	32.26±1.00ab	32.76±1.79ab	34.37±1.94a
10.98	1115	Phenylethyl Alcohol	51.24±2.20	51.41±2.39	51.68±1.92	52.07±2.85	52.59±0.71
12.61	1152	Isopulegol	0.32±0.02	0.31±0.02	0.30±0.03	0.30±0.03	0.32±0.02
12.82	1156	cis-3-Nonen-1-ol	1.47±0.08	1.48±0.16	1.51±0.27	1.54±0.21	1.61±0.07
12.94	1158	(E,Z)-3,6-Nonadien-1-ol	1.32±0.04	1.28±0.15	1.30±0.15	1.26±0.13	1.31±0.03
13.24	1164	Myrtenol	0.46±0.02a	0.42±0.02b	0.40±0.03bc	0.40±0.01bc	0.37±0.02c
13.66	1173	trans-Pyranoid linalool oxide	16.73±1.01b	17.19±1.24ab	17.24±1.10ab	18.08±1.04ab	18.78±0.36a
13.94	1178	cis-Pyranoid linalool oxide	29.23±0.78	29.46±2.46	29.48±2.43	29.56±2.25	29.78±0.25
14.43	1187	2,6-Dimethyl-3,7-octadiene-2,6-diol	1.49±0.07	1.50±0.18	1.50±0.18	1.51±0.12	1.51±0.08
14.87	1195	dihydrocarveol	1.81±0.04	1.77±0.11	1.80±0.08	1.80±0.16	1.83±0.12
16.36	1224	Nerol	3.21±0.29b	3.34±0.32ab	3.55±0.14ab	3.57±0.32ab	3.77±0.24a
16.64	1229	Isogeraniol	1.94±0.11	1.95±0.15	2.00±0.18	2.04±0.33	2.06±0.10
17.96	1253	Geraniol	26.84±0.78	26.89±1.86	27.12±1.56	27.33±1.69	27.83±1.28
19.13	1273	2,6-Dimethyl-1,7-octadiene-3,6-diol	1.82±0.15	1.85±0.11	1.85±0.15	2.08±0.11	2.15±0.25

20.77	1299	Cumic alcohol	0.32±0.04c	0.44±0.04ab	0.49±0.05a	0.40±0.02b	0.39±0.01b
23.87	1361	8-Hydroxylinalool	0.81±0.08	0.83±0.09	0.84±0.08	1.02±0.14	0.95±0.13
33.82	1555	Nerolidol	4.71±0.25a	4.36±0.48ab	4.27±0.31ab	4.25±0.60ab	3.92±0.12b
35.62	1591	Alpha-Cedrol	0.94±0.05	0.96±0.05	1.15±0.12	1.00±0.10	1.02±0.05
36.50	1616	Widdrol	0.56±0.05	0.63±0.01	0.66±0.11	0.75±0.10	0.66±0.04
37.06	1636	Cadinol T	0.36±0.01c	0.37±0.02c	0.41±0.04bc	0.52±0.05a	0.42±0.01b
37.45	1649	alpha-Cadinol	0.82±0.02	0.87±0.08	0.93±0.11	0.80±0.08	0.64±0.48
39.85	1750	cis-Lanceol	0.04±0.00b	0.05±0.00a	0.04±0.00b	0.05±0.01a	0.05±0.01a
44.58	2108	Phytol	0.50±0.01a	0.47±0.05ab	0.37±0.04c	0.30±0.03d	0.41±0.03bc
		**Alkanes & Alkenes**					
8.00	1031	Limonene	10.33±0.80b	10.40±1.00b	13.97±1.56a	12.37±1.61ab	12.27±0.75ab
15.20	1201	Dodecane	0.38±0.04b	0.38±0.01b	0.43±0.02b	0.49±0.05a	0.50±0.04a
20.93	1302	1-Methylnaphthalene	0.13±0.02ab	0.12±0.01bc	0.15±0.02a	0.10±0.01c	0.11±0.02bc
25.97	1398	Tetradecane	0.36±0.02	0.35±0.02	0.37±0.06	0.40±0.07	0.41±0.01
29.15	1461	Alloaromadendrene	0.03±0.00c	0.04±0.00bc	0.05±0.01a	0.04±0.01b	0.04±0.00bc
31.08	1496	α-Farnesene	0.61±0.06c	0.85±0.03bc	1.01±0.17b	1.56±0.08a	1.59±0.23a
31.60	1505	δ-Cadinene	0.81±0.06b	0.83±0.06b	0.97±0.06a	0.82±0.01b	0.75±0.04b
		**Esters**					
14.64	1191	Methylsalicylate	12.25±0.88	12.32±0.92	12.82±0.90	12.91±0.99	12.92±0.80
16.58	1228	Linalylformate	1.28±0.03	1.28±0.05	1.27±0.13	1.28±0.13	1.30±0.09
17.11	1238	cis-3-Hexenyl3-methylbutanoate	2.97±0.16	2.95±0.18	2.96±0.14	3.12±0.29	3.20±0.45

19.43	1278	1,5-Octalactone	1.88±0.05	1.96±0.17	1.99±0.14	2.04±0.08	2.38±0.35
19.81	1284	Isopulegylacetate	0.24±0.03a	0.21±0.02a	0.21±0.04a	0.15±0.02b	0.20±0.02a
23.65	1356	Gamma-Nonalactone	0.76±0.11	0.77±0.11	0.79±0.05	0.90±0.07	0.85±0.12
24.06	1364	Butoxyethoxyethylacetate	2.74±0.17	2.83±0.15	3.16±0.23	3.06±0.30	2.90±0.15
24.91	1380	cis-3-Hexenylhexanoate	6.73±0.29	6.46±0.26	6.22±0.93	6.17±0.75	5.75±0.40
25.09	1383	cis-3-Hexenylcis-3-hexenoate	1.33±0.14a	0.96±0.11b	0.90±0.06b	1.03±0.16b	0.93±0.11b
25.19	1385	Hexylhexanoate	2.76±0.08	2.79±0.14	2.86±0.26	3.03±0.28	2.73±0.18
25.37	1388	trans-2-Hexenylhexanoate	5.45±0.20a	4.51±0.27b	4.49±0.29b	4.48±0.54b	4.27±0.30b
27.05	1420	Coumarin	0.80±0.09	0.82±0.04	0.86±0.04	0.92±0.07	0.92±0.09
27.58	1431	Phenethylbutyrate	0.50±0.02c	0.51±0.03c	0.52±0.02bc	0.55±0.02b	0.64±0.02a
29.98	1476	Jasminelactone	1.41±0.13	1.40±0.07	1.37±0.11	1.39±0.16	1.43±0.09
30.16	1480	δ-Amylvalerolactone	0.39±0.01c	0.45±0.03ab	0.42±0.04bc	0.38±0.02c	0.48±0.00a
31.71	1509	Dihydroactinidiolide	1.37±0.06	1.38±0.07	1.42±0.14	1.45±0.12	1.57±0.06
32.98	1537	BenzylHexanoate	0.32±0.01a	0.23±0.01b	0.25±0.04b	0.24±0.02b	0.24±0.01b
34.16	1562	cis-3-Hexenylbenzoate	1.96±0.16	1.94±0.15	2.02±0.22	1.93±0.24	1.98±0.05
34.57	1570	Hexylbenzoate	0.32±0.02	0.32±0.05	0.36±0.03	0.33±0.02	0.35±0.02
34.95	1578	trans-2-Hexenylbenzoate	0.58±0.03	0.60±0.05	0.66±0.07	0.60±0.06	0.61±0.03
35.50	1589	Caryophyleneoxide	0.30±0.04	0.30±0.01	0.31±0.06	0.29±0.02	0.32±0.02
39.31	1720	Methylmyristate	0.18±0.02	0.18±0.01	0.18±0.01	0.18±0.02	0.17±0.02
41.52	1854	Diisobutylphthalate	0.34±0.01	0.36±0.02	0.40±0.05	0.38±0.01	0.40±0.03
42.45	1924	Methylhexadecanoate	2.05±0.07	2.06±0.17	2.01±0.11	2.00±0.04	2.01±0.12

42.79	1952	Dibutylphthalate	0.09±0.01	0.10±0.02	0.10±0.02	0.10±0.01	0.12±0.02
44.46	2098	Methyloleate	0.19±0.01	0.18±0.03	0.18±0.01	0.18±0.03	0.19±0.01
		**Aldehydes & Ketones**					
4.16	854	(E)-2-Hexenal	11.70±0.11	11.93±0.77	12.26±0.43	12.53±2.17	13.17±1.27
6.04	956	(E)-2-Heptenal	0.15±0.01	0.14±0.02	0.17±0.02	0.17±0.01	0.17±0.01
6.21	963	Benzaldehyde	3.79±0.24	3.84±0.16	4.08±0.26	4.18±0.61	4.41±0.31
7.43	1012	(E,E)-2,4-Heptadienal	1.05±0.05b	1.07±0.03b	1.30±0.11a	1.34±0.19a	1.39±0.16a
8.43	1045	Phenylacetaldehyde	28.51±1.02	28.41±1.45	28.66±2.67	29.21±3.66	31.87±1.06
8.88	1059	(E)-2-Octenal	1.26±0.11b	1.29±0.21b	1.88±0.14a	1.82±0.03a	1.67±0.20a
9.22	1069	Furaneol	2.95±0.06a	2.93±0.15a	2.86±0.23a	2.26±0.33b	1.62±0.23c
13.13	1162	(E)-2-Nonenal	0.78±0.05	0.76±0.09	0.82±0.09	0.82±0.03	0.88±0.05
15.00	1197	Safranal	1.83±0.11	1.71±0.14	1.71±0.23	1.63±0.15	1.65±0.19
15.44	1206	Decanal	1.06±0.14	1.06±0.15	1.07±0.14	1.09±0.10	1.11±0.16
15.85	1214	(E,E)-2,4-Nonadienal	0.60±0.02c	0.83±0.02bc	1.05±0.17ab	1.07±0.20ab	1.09±0.19a
16.05	1218	β-Cyclocitral	7.92±0.18b	8.02±0.21b	8.54±0.56b	8.59±0.58ab	9.40±0.58a
18.06	1255	2,6,6-Trimethyl-1-cyclohexene-1-acetaldehyde	2.55±0.09	2.53±0.22	2.54±0.27	2.55±0.03	2.53±0.20
18.58	1264	trans-2-Decenal	0.95±0.10	0.96±0.14	0.98±0.15	0.99±0.13	1.13±0.09
18.77	1267	α-Ethylidenbenzeneacetaldehyde	6.98±0.19a	5.69±0.21bc	5.88±0.27bc	5.91±0.18b	5.54±0.14c
18.86	1269	Citral	1.37±0.12c	1.31±0.08c	1.35±0.05c	1.53±0.04b	1.70±0.09a
20.57	1296	Perillaldehyde	0.23±0.02	0.25±0.01	0.25±0.02	0.24±0.02	0.26±0.02
21.65	1317	(E,E)-2,4-Decanedienal	1.59±0.18	1.53±0.22	1.49±0.11	1.33±0.08	1.33±0.12

24.63	1374	Damascenone	1.68±0.14	1.75±0.06	1.87±0.09	1.76±0.14	1.67±0.08
25.32	1387	Jasmone	1.56±0.08bc	1.44±0.10c	1.89±0.23a	1.88±0.11a	1.81±0.15ab
26.70	1413	Alpha-Ionone	1.39±0.09	1.42±0.17	1.51±0.10	1.60±0.19	1.69±0.19
28.07	1440	Nerylacetone	1.06±0.08	1.05±0.14	1.02±0.15	0.99±0.14	0.98±0.16
29.53	1468	β-Ionone	9.40±0.09	8.51±0.99	8.48±1.12	8.34±0.81	8.44±0.72
29.69	1471	β-Ionone-5,6-epoxide	1.61±0.19b	1.67±0.12b	1.83±0.14ab	1.85±0.10ab	1.98±0.07a
29.84	1474	5-Methyl-2-phenylhex-2-enal	1.49±0.06	1.51±0.19	1.51±0.14	1.50±0.13	1.52±0.14
		**Acids**					
6.87	991	Hexanoic acid	2.47±0.30c	2.68±0.45c	2.79±0.96c	3.84±0.28b	4.90±0.49a
7.38	1010	(E)-3-hexenoic acid	10.33±1.19	10.41±0.35	10.52±0.70	10.95±0.67	11.84±0.86
9.70	1082	Heptanoic acid	0.77±0.12	0.79±0.03	0.93±0.15	0.87±0.05	0.84±0.12
19.04	1272	Nonoic acid	2.49±0.04c	2.44±0.12c	2.31±0.14c	2.88±0.30b	3.39±0.19a
23.39	1351	Geranic acid	1.48±0.09	1.40±0.21	1.39±0.13	1.37±0.19	1.36±0.25
		**Phenols**					
20.14	1289	Thymol	1.07±0.05	1.06±0.02	1.08±0.04	1.07±0.02	1.08±0.06
23.26	1349	Eugenol	2.88±0.27ab	3.11±0.07a	2.59±0.21bc	2.42±0.18c	2.67±0.11bc
30.88	1492	Butylated Hydroxytoluene	0.26±0.02b	0.22±0.03b	0.15±0.02c	0.36±0.01a	0.37±0.00a
31.18	1498	2,4-Di-t-Butylphenol	0.97±0.07a	0.96±0.05a	0.69±0.03b	0.67±0.05b	0.72±0.03b
		**Others**					
9.14	1067	2-Acetylpyrrole	nd	nd	1.84±0.17a	1.43±0.10b	1.45±0.08b
20.07	1288	Indole	1.25±0.05a	1.21±0.02a	1.10±0.03b	1.07±0.02b	1.08±0.00b

21.11	1306	Edulane	nd	nd	0.99±0.07a	0.82±0.01b	0.89±0.05b
21.32	1310	2,6,10,10-Tetramethyl-1-oxaspiro[4.5]dec-6-ene	0.40±0.02c	0.66±0.03a	0.53±0.05b	0.39±0.04c	0.40±0.01c

Note: nd indicates that the substance was not detected in the samples; different small letters in the same row indicate significant difference at $p < 0.05$.
